# Social Identity Threat Motivates Science-Discrediting Online Comments

**DOI:** 10.1371/journal.pone.0117476

**Published:** 2015-02-03

**Authors:** Peter Nauroth, Mario Gollwitzer, Jens Bender, Tobias Rothmund

**Affiliations:** 1 Philipps-University Marburg, Marburg, Germany; 2 University of Koblenz-Landau, Landau, Germany; University of Western Brittany, FRANCE

## Abstract

Experiencing social identity threat from scientific findings can lead people to cognitively devalue the respective findings. Three studies examined whether potentially threatening scientific findings motivate group members to take action against the respective findings by publicly discrediting them on the Web. Results show that strongly (vs. weakly) identified group members (i.e., people who identified as “gamers”) were particularly likely to discredit social identity threatening findings publicly (i.e., studies that found an effect of playing violent video games on aggression). A content analytical evaluation of online comments revealed that social identification specifically predicted critiques of the methodology employed in potentially threatening, but not in non-threatening research (Study 2). Furthermore, when participants were collectively (vs. self-) affirmed, identification did no longer predict discrediting posting behavior (Study 3). These findings contribute to the understanding of the formation of online collective action and add to the burgeoning literature on the question why certain scientific findings sometimes face a broad public opposition.

## Introduction

“Another simple pseudo-scientist who gets a pat on the back for finding what he was looking for. No subtle thinking here. No qualifying or consideration of alternate interpretation. No honest presentation of the limits of your study. No alternative explanations. This is why the majority of social scientists are flimsy. It is a weak science desperately pretend it has hard evidence for complex phenomena.”

The Internet has changed the way we communicate and engage with each other, and it has also changed the way scientists communicate their findings to the general public as well as how laypersons inform themselves and engage with science. Before the Internet era, science communication was mostly indirect: newspapers, magazines, and TV shows reported about science, and the public had little chance to actively engage with scientists and their research. This has changed. Science blogs, discussion forums, podcasts, and video channels give scientists the opportunity to inform and discuss their research directly with people all over the world. These new possibilities were enthusiastically greeted by scientists across diverse disciplines [1] and many of them actively use the new communicational features provided by the Internet to inspire the public or even to acquire public funding.

As always, new communicative possibilities not only promise opportunities, they also involve problems. Just as it was never easier to publicly promote science, it was also never easier to publicly discredit it. Blogs, discussion forums, or newspaper websites allow people to directly evaluate and devaluate scientific findings they read about online. Plausibly, the distribution of praise and criticism in publicly visible online comments to a particular scientific enterprise has consequences for the general public’s impressions about the quality of the respective research [2] and also about the risks associated with the scientific enterprise [3]. Traditionally, critiques about research were voiced in commentary sections of journals in which a finding was published. These commentaries were also peer-reviewed, which was an attempt to secure a certain degree of qualification. This is not the case with online comments. For instance, a YouTube video produced by a prominent researcher on the effects of violent video games [4] yielded 185 ratings (as of March 31, 2014). The vast majority of these ratings (86%) were negative (i.e., the video was “disliked”), and 85 out of 97 users severely criticized the author’s research fundamentally, attacked his scientific reputation, and offended him personally by posting a negative comment. The statement quoted at the beginning of this article is one of these comments. This example illustrates that the Internet, especially “Web 2.0” features, open the stage for a public devaluation of science by anybody who is motivated to do so. The consequences of this particular form of “public engagement with science” can be profoundly negative [3].

One might argue that we are overly pessimistic, and that cases in which scientific findings face a notable public opposition are rare. Unfortunately, this is not the case. Misinformation concerning scientific findings is widespread, and the Internet enables an effective dissemination of these false beliefs [5]. Anthropogenic climate change, evolutionary theory, side-effects of vaccines, effectiveness of alternative medicine, or the effects of violent video games: all these issues were controversially and heatedly discussed in the public. The question what motivates people to publicly discredit scientific findings and to disseminate mistrust in science on the Web is therefore of great societal interest and importance.

So far, science communication research has identified several factors that may promote a critical evaluation of research results. For instance, findings that call long-held values, beliefs, or attitudes into question are evaluated more negatively than results confirming these beliefs [6–9]. Notably, most of the research on science communication has focused on how *individually* held attitudes or beliefs influence people’s reception of science. However, scientific research sometimes affects social or societal groups–either explicitly or implicitly. For example, research on the consequences of a vegetarian diet affects the group of vegetarians; research on men’s sexist attitudes against women affects the group of men; and research on the effects of playing violent video games affects the group of video game players (who are henceforth referred to as “gamers” for reasons of simplicity). Recent evidence suggests that whenever research affects entire groups, social identity concerns play an important role in the reception of scientific findings [10–12]. For instance, Nauroth and colleagues [11] showed that gamers’ evaluation of research about the negative effects of playing violent video games was best explained by their identification with the group of gamers over and above their personal beliefs about the effects of violent video games and their gaming habits. These results indicate that social factors, such as group membership and identification with one’s group, are crucial in the reception of scientific findings as soon as these findings directly or indirectly affect the respective group.

Although much research has focused on factors that explain a biased *evaluation* of scientific findings, virtually no research has addressed the *behavioral outcomes* of these evaluations so far. This is remarkable since holding a negative attitude against research must not necessarily translate into action against the respective research [13]. It therefore remains an open question whether the effects found with regard to the evaluation of scientific findings have also downstream behavioral consequences. At the societal level, understanding such behavioral consequences, that is, publicly discrediting or even actively opposing science, are highly relevant, since they can substantially impair positive societal change.

The present research aims to fill this gap: based upon social identity theory [14,15], the social identity model of deindividuation effects [16] and research on collective action [17], we argue that social identification and social identity threat can explain why people negatively evaluate *and* publicly oppose scientific findings. Whenever research findings negatively affect a certain group, we hypothesize that publicly criticizing such research on the Web is motivated by a perceived social identity threat and directed at defending the group’s image and one’s social identity.

### Social Identification Motivates Collective Action (Against Science)

How group-relevant information is interpreted strongly depends on whether or not people categorize themselves as members of the respective group [18,19]. Thus, a scientific study should be evaluated more positively when it affirms one’s social identity. In line with this notion, Morton and colleagues [10] showed that participants considered a study to be more “scientific” and were more interested in this research when it affirmed their gender identity. They concluded that scientific findings are more likely to be perceived as credible and plausible when they provide people with a positive sense of identity, irrespective of the actual scientific state of the art. In addition, the degree to which people identify with a group qualifies the effect of group membership on social information processing: strongly identified group members are more likely to show a biased evaluation than weakly identified members when the information is threatening to their respective social identity [20,21]. In line with this argument, Nauroth and colleagues [11] showed that strongly (vs. weakly) identified gamers were more likely to devalue research on the detrimental effects of violent video games to the extent that this research constituted a social identity threat.

Rejecting and devaluing science in private is one thing, but criticizing science and the scientists behind it in public is something different, and not much is known about the social and cognitive dynamics underlying this detrimental form of a “public engagement with science.” Thus, a look into the literature on behavioral responses towards social identity threat might be helpful here. A first important finding from this literature is that, in the face of a social identity threat, strong identifiers show solidarity toward the in-group by taking action against the threat (e.g., protesting). For instance, Scheepers and colleagues [22] demonstrated that strong identifiers are more likely than weak identifiers to insult the out-group when the in-group was threatened. According to Scheepers and colleagues, the instrumentality of such out-group derogation is to direct in-group members’ attention and effort toward group-relevant goals and to motivate collective action. In the same vein, Van Zomeren and colleagues [17] provided evidence for the importance of social identification for collective action in a comprehensive meta-analysis. These authors investigated perceived injustice, perceived efficacy, and social identification as potential psychological determinants of collective action tendencies. Results showed that social identification did not only promote collective action directly but also indirectly via perceived injustice and perceived efficacy. Thus, there is evidence that social identification predicts not only a biased evaluation of threatening information but also (collective) social action against a social identity threat. However, there is virtually no research on the predictive value of social identification in the area of science communication. Based on these arguments and findings, we hypothesize that strongly identified group members should be more inclined to actually show science-discrediting behavior against a threatening scientific study.

The empirical evidence on social identity and collective action reviewed so far only investigated non-online behavior. However, there is reason to assume that these effects also apply to behavior in the online realm. The SIDE model of computer-mediated communication effects proposes that visual anonymity (as on the Web) makes it difficult to discern individuals’ interpersonal differences and, thereby, depersonalizes self- and other-perception [16]. When a social identity is salient, visual anonymity should amplify social identity influences on perceptions and behavior [23]. For example, Postmes and colleagues [24] showed that visual anonymity leads to a stronger identification with the in-group and a greater consensus with the in-group’s norms. Thus, social identity effects, as predicted by social identity theory and research on collective action, should even be more pronounced on the Web when a social identity is already salient [25]. Applied to the case in which strongly identified group members read an article presenting potenially threatening research results on a news site, anonymity should even enhance their identification and, in turn, they should be more inclined to evaluate the research findings negatively and react upon the article with a harsh, offensive and science-discrediting comment.

### Acting Against Science as a Means to Restore Social Identity

Even though it is theoretically plausible to assume that social identification motivates science-discrediting comments, the psychological mechanism underlying this effect is not clear. Basing our research on the social identity approach [14,15], we conceptualize social identity as aspect of the overall self-concept that is independent from personal identity. *Personal identity* refers to people’s self-definition as unique and different from other individuals. *Social identity*, by contrast, refers to the part of the self-concept being defined by one’s affiliation to social categories. In the context of an identity threat emanating from scientific research this distinction becomes important because it has immediate consequences on one’s reactions to the threat and the motivations underlying these reactions. A research finding indicating the harmfulness of video game violence might not only pose a threat to one’s social identity but also to one’s personal identity. For example, strongly identified gamers could define themselves as being competent users of digital media. Thus, group members, particularly strong identifiers, may feel personally attacked by this research experiencing an ego-threat [26]. Posting a science-discrediting comment could then be understood as an ego-defense mechanism. In other words, even though posting negative comments is predicted by social identification, it may not so much be a collective, but rather a personal issue.

Research on self-affirmation theory [27] has shown that defensive reactions toward threatening information are less likely when individuals are given the opportunity to affirm their personal identity [28]. For example, Toma and Hancock [29] demonstrated that participants were more receptive toward negative performance feedback when they wrote a short essay about a value that is important to them (“self-affirmation”). Notably, this was also the case when participants were allowed to spend time on their Facebook profile, demonstrating that personal identity concerns play an important role in explaining online behavior. Thus, if the mechanism underlying negative posting was motivated by personal identity concerns, self-affirming group members should lead them to refrain from posting a science-discrediting comment.

By contrast, posting negative comments might rather be genuinely motivated by the desire to affirm one’s *social* identity. Since the self-concept of strong identifiers is more strongly based on their group membership [15,20] they should also have a higher motivation to restore their social identity [30]. Thus, acting against a social identity threat may rather aim to affirm one’s social identity [31,32]. This reasoning is in line with research showing that strongly identified group members tend to choose social identity affirmative strategies after being threatened [33]. Since collective action is a social identity affirmative strategy [15] posting a science-discrediting comment on the Internet might also serve as a means to restore one’s social identity after it has been threatened by the respective scientific finding.

If posting a negative comment was indeed motivated by the desire to reaffirm one’s social identity, alternative ways of affirming one’s social identity—for instance, shifting one’s attention on the positive aspects of one’s group—should therefore reduce the likelihood of posting a negative comment about potentially threatening research. This argument resonates with recent findings showing that alluding group members to positive group characteristics increases their acceptance of potentially threatening information. For instance, Sherman and colleagues [32] demonstrated that strongly identified university sports team fans were particularly likely to exhibit group serving attributions after their team lost. However, this bias was alleviated when strongly identified fans were asked to think and write about important values of their university. In other words, giving group members the opportunity to reflect upon positive aspects of their group (“collective affirmation”) may act as a shield against potentially threatening information. This implies that collectively affirming group members should alleviate their desire to act against science as a means to restore their social identity, for instance, by posting science-discrediting online comments. If our reasoning was true and posting negative comments was a collective rather than a personal issue, a personal self-affirmation should be less effective than a group-based “collective” affirmation. Thus, we hypothesize that collectively affirmed strong identifiers should be less likely to post a harsh devaluing comment about a potentially threatening scientific finding than non-affirmed strong identifiers. Personal self-affirmation, however, should not have such an effect.

### The Present Research

In the present research we focus on the violent video games debate in order to test our predictions. Potential negative effects of playing violent video games have been fiercely discussed over the last 15 years, not only outside the academic world, but inside as well [34,35]. The question of whether or not violent video games have detrimental effects is still vivid not only in the public media, but also in juridical processes [36]. Not surprisingly, the public opinion on whether violent video games have detrimental effects is divided [37]. Notably, this topic is particularly suited to investigate group influences on the evaluation of scientific findings since video game players–“gamers”–are primarily affected by the research conducted on violent video games, and most people are familiar with the topic [38].

The present article describes three studies designed to test two hypotheses: The *first* hypothesis is that strongly identified gamers are more likely to act against potentially threatening research results by writing (negative) online comments targeting the credibility of the research (Studies 1, 2, and 3). The *second* hypothesis is that this effect can be alleviated when gamers are collectively (but not personally) affirmed after being threatened (Study 3). Thus, Study 3 aims at elucidating the psychological mechanism underlying the effects found in Studies 1 and 2.

Studies 2 and 3 were conducted as part of a larger research project for which a global ethics approval was obtained from the psychology department’s ethics committee at Philipps University Marburg (AZ: 2011-02K). Study 1 was conducted as part of a research project for which no ethics approval was required, neither from the funding agency nor from the department. However, all three studies were conducted in full accordance with (1) the declaration of Helsinki and (2) the ethics guidelines of the German Psychological Society. This includes–among others–obtaining informed consent, the right to withdraw at any time, and data protection. At the beginning of each study, prospective participants read detailed information regarding ethical guidelines (i.e., that the data are analyzed anonymously, that they are free to refrain from participation in the study and to withdraw consent to participate at any time without disadvantage) and had to give informed consent to this information before being able to participate in the studies. All collected information that could have made identification of participants possible were deleted before analysis (i.e., e-mail addresses collected for the raffles).

## Study 1

Study 1 provides a first test of the hypothesis that the extent to which people identify with the group of gamers positively predicts (self-reported) posting behavior when the research corroborates the violent-games-effect hypothesis (i.e., demonstrates that violent video games have detrimental effects).

### Method

We reanalyzed data collected by Sjöström and colleagues [38], who investigated audience judgments of social sciences in the violent video games debate. They conducted an Internet-based questionnaire and recruited participants based upon a representative distribution of sex, age, level of education, and state of residence in Germany. A correlational design was used to examine the relationship between social identification and (self-reported) posting behavior under social identity threat and the absence of social identity threat.

**Participants**. Sjöström and colleagues sample consisted of 290 respondents with different levels of experience in playing video games. However, for the present article we were only interested in gamers, that is, people who play video games on a regular basis. Thus, 206 participants (i.e., 71%) were excluded from further analyses because they indicated that they do not regularly play video games (i.e., at least two hours per week; *n* = 194) or indicated that they were not able to assess the state of research (a crucial variable for the present research, see below; *n* = 12). Therefore, the final sample consisted of 84 participants (46% women). Ages ranged between 18 and 62 years (*M* = 37.06; *SD* = 11.85).

**Materials and Measures**. First, *identification* with the group of gamers was measured with an adapted version of Leach and colleagues [39] social identification scale, addressing the self-investment factor of social identification (10 items, e.g., “I am glad to be a gamer;” Cronbach’s α = .92). We replaced the item “The fact that I am a gamer is an important part of my identity” with “When somebody criticizes gamers, it feels like a personal insult”. We included this item because it best measures a person’s experience of the group as an important part of the self (see [39], p. 817). Notably, Leach and colleagues [39] (p. 151 and 165) also considered including a similar item (“I feel (personally) implicated when [In-group] people are criticized.” see [41]) in their scale, but excluded it as being too vaguely formulated (which is true due to the term “implicated”). However, the wording of the item from Mael and Tetrick [40] is specific and it clearly measures self-group merging as an important facet of social identification (for a similar argument concerning this item see also [41], p. 91). That this is the case is also confirmed by the results of our scale analysis showing an adequate item discrimination of *r*_*it*_ = .46 (when omitting this item, the identification × current state of research interaction still predicted posting behavior, *B* = 0.19, *SE(B)* = 0.09, *p* = .04, ∆*R^2^* = .05). Ratings were obtained on a six-point scale (1 = *strongly disagree*, 6 = *strongly agree*).

Next, the *assessment of the current state of violent video games research* was measured with one item (“According to your opinion, to what extent does the following statement apply to the current state of research: On average, violent video games increase aggressive thoughts, feelings, and behavior in the long term.”). Responses were made on a five-point scale (-2 = “*the current state of research clearly contradicts this statement*,” 2 = “…*clearly supports this statement*”) with the additional option to indicate that one is not able to assess the state of research. Therefore, positive values indicate participants’ assumption that the current state of research shows that violent video games cause aggression, and negative values indicate that this is not the case.

Next, *posting behavior* was assessed with two items (“I comment on articles about violent video games on web portals (e.g., Spiegel Online),” and “I engage in discussions about violent video games in forums and blogs;” Cronbach’s α = .90). Responses were made on a six-point scale (1 = *never*, 6 = *very often*). Descriptive statistics and correlations between all variables are displayed in Table 1. Identification with the group of gamers and assessment of the current state of research were negatively correlated (*r* = −.32), which (plausibly) implies that strongly identified gamers were less likely to believe that the current research corroborates the violent-games-effect hypothesis compared to weakly identified gamers. Notably, however, this correlation was of medium size, which makes it possible to scrutinize the identification × current state of research interaction effect on posting behavior.

**Table 1:** Descriptive Statistics and Correlations between Variables in Study 1.

Variable	M (SD)	Correlations		
		(1)	(2)	(3)
Identification with the group of gamers (1)	2.63 (1.06)	1.00		
Assessment of the current state of research (2)	0.40 (1.20)	−0.32**	1.00	
Posting behavior (3)	1.92 (1.17)	0.21	−0.02	1.00

*Notes*. *N* = 84.

*p < .05

**p < .01

***p < .001.

### Results

**Figure 1:**
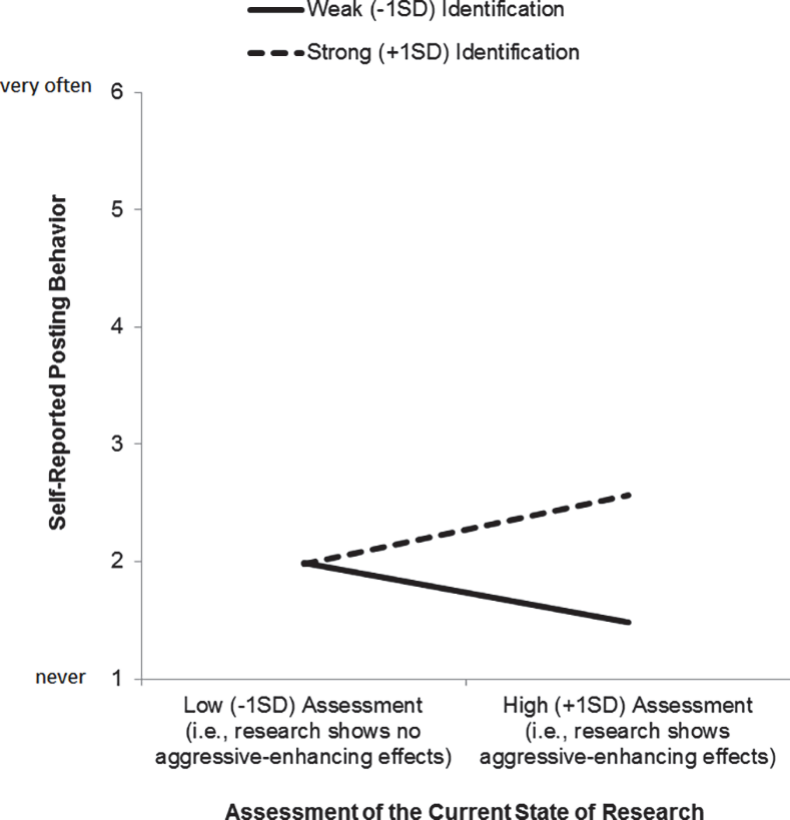
Self-reported posting behavior by identification with the group of gamers and assessment of the current state of research (Study 1).

We tested our hypothesis via moderated regression analysis [42] and centered identification and assessment of the current state of research prior to computing product terms [43]. As expected, self-reported posting behavior was predicted by the identification × current state of research interaction, *B* = 0.21, *SE*(*B*) = 0.09, *p* = .03, ∆*R* ^2^ = .06. Simple slopes analyses revealed that the effect of current state of research was larger for strongly identified gamers (i.e., 1 *SD* above the sample mean), *B* = 0.24, *SE*(*B*) = 0.14, *p* = .08, than for weakly identified gamers (i.e., 1 *SD* below the sample mean), *B* = −0.21, *SE*(*B*) = 0.16, *p* = .19. Additionally, we found a main effect of identification, *B* = 0.26, *SE*(*B*) = 0.12, *p* = .04, indicating that strongly identified gamers were on average more likely to comment on research on violent video games. The main effect of assessment was not significant, *B* = 0.02, *SE*(*B*) = 0.11, *p* = .88. Predicted means are displayed in Fig. 1.

### Discussion

The results of Study 1 provide first evidence for our notion that posting behavior is influenced by the degree to which gamers identify with their in-group. This finding supports our first hypothesis that potentially threatening research triggers behavioral responses in the form of online comments among strongly identified group members.

Although the results of Study 1 generally support our predictions, there are some limitations and shortcomings. Firstly, the items measuring posting behavior were rather unspecific. With these items we have no information about the valence of the posted comments, only about the frequency. We know that strongly identified group members are more likely to comment on research about violent video games if they perceive research to confirm negative effects. However, we do not know whether strongly identified group members are more likely to post *discrediting* comments about threatening research. Secondly, we did not assess behavior directly, but merely relied on self-reports. Frequency self-reports are often biased and not an accurate assessment of the true behavior frequency [44]. Assessing posting behavior directly would certainly provide a better test of our first hypothesis. Finally, and most importantly, we used a correlational design to test our predictions. Possibly, the assessment of the current state of research is confounded by other variables not included in our study, such as a critical attitude towards science in general. In order to remedy these shortcomings, participants in Study 2 were asked to write a negative or positive blog comment about a threatening or non-threatening scientific study. Furthermore, Study 2 used an experimental approach in order to strengthen the internal validity of the design.

## Study 2

Study 2 aimed at replicating the effect of identification on posting behavior. However, in contrast to Study 1, we used an experimental approach and a behavioral measure of posting behavior, which is able to discern between negative and positive comments. Participants could actually write a “pro” or “contra” comment about two scientific studies, one corroborating (“confirmatory” study) and one disconfirming (“confutative” study) the violent-games-effect hypothesis. Importantly, besides investigating the likelihood of writing a positive (i.e., “pro”) or a negative (i.e., “contra”) comment, we also content-analyzed all posted comments with regard to their evaluative and opinionative statements. We expected that strongly (vs. weakly) identified gamers were more likely to write a negative comment about the confirmatory study, but not about the confutative study.

Additionally, we implemented a blog environment in order to increase the ecological validity with the possibility to use a “like” or “dislike” button often found on the Internet. “Like” and “dislike” buttons provide an efficient way to indicate one’s approval or disapproval of content on websites [45]. Therefore, these buttons constitute another opportunity for motivated group members to influence the public opinion about the featured research. Furthermore, we assessed participants’ evaluation of the respective studies [11]. We expected that strongly (vs. weakly) identified gamers dislike the confirmatory study more often and evaluate the confirmatory study more negatively than the confutative study.

### Method

Participants. Participants were recruited via an internal university student mailing list. In all, 976 people started the questionnaire; 705 of them (72%) finished it successfully. Fifty participants were excluded from further analyses because these people indicated that they had not played any video games during the last 12 months (*n* = 33) or had more than 25% missing values on one or more scales (*n* = 17). Therefore, the final sample consisted of 655 participants (34% women). Ages ranged between 18 and 51 years (*M* = 23.64; *SD* = 3.30). One tablet computer was raffled among all participants who completed the survey.

**Design and Procedure**. Study 2 was conducted as an online-based experimental survey using a one-factorial within-subjects design. Participants were told that a new blog about recent research findings was going to be launched soon, and that the first topic to be discussed in this new blog would be “violent video games.” The survey would be conducted in order to identify studies suitable to be posted in the blog. Participants were instructed to read short summaries from two studies which were ostensibly randomly chosen from a larger pool of studies, to rate each study by “liking” or “disliking” it, and to comment on each study in the blog if they wished to do so (for a design overview see Table 2).

**Table 2:** Design of Study 2.

Condition	Confirmatory Study		Confutative Study	
Liking/Disliking	“thumb up”-button (i.e., like)	“thumb down”-button (i.e., dislike)	“thumb up”-button (i.e., like)	“thumb down”-button (i.e., dislike)
Posting Behavior	“Pro comment” textbox (i.e., positive comment)	“Contra comment” textbox (i.e., negative comment)	“Pro comment” textbox (i.e., positive comment)	“Contra comment” textbox (i.e., negative comment)
Content Analysis	Frequency of 5 categories		Frequency of 5 categories	
Evaluation	Evaluation of the study (9 items)		Evaluation of the study (9 items)	

One of the two studies found empirical evidence for the hypothesis that playing violent video games has detrimental effects (“confirmatory study” condition) whereas the other refuted this hypothesis empirically (“confutative study” condition). In order to reduce suspicion and to enhance credibility, the two “studies” differed in their methodologies: One of them was said to have used a behavioral measure of aggression as the central dependent variable; the other used fMRI data (see S1 Appendix). The order in which the two studies (more precisely, the two experimental conditions) were presented as well as their respective methodologies was fully counterbalanced across participants. All patterns of results remained the same when the order and type of the manipulation studies and their interactions with identification where included in the analyses of posting behavior, liking/disliking behavior, and biased evaluation. Importantly, all interaction terms including identification (i.e., identification × order, identification × type, identification × order × type) were not significant on the 5% level.

**Identification**. First, identification with the group of gamers was measured with an adapted 5-item version of the Leach and colleagues [39] subscale of the self-investment factor. Additionally, we included one item to measure identification with the group of gamers on a broader level adapted from Postmes, Haslam, and Jans [46] (“I identify with the group of ‘gamers’”). This six-item measure constituted a reliable scale (Cronbach’s α = .92). Ratings were obtained on a six-point scale (1 = *strongly disagree*, 6 = *strongly agree*).

**Liking/Disliking Behavior**. Then, participants read the two study summaries and indicated whether they *liked* or *disliked* the respective study by clicking on a thumbs-up or thumbs-down shaped button below the respective summary. We were not able to collect data on liking/disliking from 10 participants because they had JavaScript deactivated in their browser. Clicking on a button was not mandatory; thus, there were three possible responses to each study: like, dislike, and no response. Table 3 shows how response patterns were coded for subsequent analyses. According to this coding scheme, response patterns in which (a) the confirmatory study (but not the confutative study) was disliked or those in which (b) the confutative study (but not the confirmatory study) was liked were coded as +1, indicating a *disliking bias against the confirmatory study*; patterns in which (a) the confirmatory study (but not the confutative study) was liked or those in which (b) the confutative study (but not the confirmatory study) was disliked were coded as –1, indicating a *liking bias in favor of the confirmatory study*. All remaining patterns (i.e., both studies were liked, both studies were disliked, or neither study received a response) were coded as 0. This coding scheme corresponds to our conceptual hypotheses: it directly compares those participants who remained neutral towards either study (coded with 0) against those who favor the confutative over the confirmatory study (coded with +1) and against those who favor the confirmatory over the confutative study (coded with –1).

**Table 3:** Dependent Variable Coding Scheme for Liking/Disliking Behavior (Study 2).

		Confirmatory Study			
		like	no response	dislike	total
Confutative Study	like	0	1	1	
		(91)	(25)	(181)	(297)
	no response	−1	0	1	
		(8)	(139)	(11)	(158)
	dislike	−1	−1	0	
		(91)	(7)	(92)	(190)
	total	(190)	(171)	(284)	(645)

*Notes*. *N* = 655. Number of participants in each category in parentheses.

**Comments**. On the same site below the like/dislike buttons, participants found two text boxes labeled “PRO comments” and “CONTRA comments.” Participants had the opportunity to write a “pro” (positive) and/or a “contra” (negative) comment toward each study in the respective text box. Table 4 shows how response patterns were coded: patterns in which participants commented more *negatively* on the confirmatory than on the confutative study (and more positively on the confutative than on the confirmatory study) were coded as +1, indicating a *posting bias against the confirmatory study*; patterns in which participants commented more *positively* on the confirmatory than on the confutative study (and more *negatively* on the confutative than on the confirmatory study) were coded as –1, indicating a *posting bias in favor of the confirmatory study*; patterns in which none of the studies were commented on or in which both studies received similar comments were coded as 0 (no bias). Again, this coding scheme corresponds to our conceptual hypotheses: it directly compares those participants who did not favor either study in their comments (coded with 0) against those who criticize the confirmatory study more than the confutative study (coded with +1) and against those who praise the confirmatory more than the confutative study (coded with –1). This coding scheme directly contrasts the two studies with regard to participants’ commenting behavior. One anonymous reviewer pointed out that it would also be worthwhile to analyze participants’ commenting behavior separately for each study. This analysis is reported in S1 and S2 Tables. Besides counting the number of “pro” and “contra” comments per article, these comments were also content-analyzed (see below).

**Table 4:** Dependent Variable Coding Scheme for Posting Behavior (Study 2).

		Confirmatory Study				
		only positive	no comment	positive & negative	only negative	Total
Confutative Study	only positive	0	1	1	1	
		(53)	(5)	(6)	(154)	(218)
	no comment	−1	0	0	1	
		(11)	(123)	(1)	(22)	(157)
	positive & negative	−1	0	0	1	
		(5)	(4)	(28)	(20)	(57)
	only negative	−1	−1	−1	0	
		(89)	(11)	(18)	(105)	(223)
	total	(158)	(143)	(53)	(301)	(655)

*Notes*. *N* = 655. Number of participants in each category in parentheses.

**Evaluation**. After commenting on one summary, participants were asked to evaluate the respective research (including the authors) on nine items. Seven items were taken from Nauroth and colleagues [11] (“I think that this study was a waste of public money,” “I think that these authors are not very competent,” “I think that the results of this study are unambiguous” (recoded), “I think that these authors just find what they wanted to find,” “I think that this study yielded important results” (recoded), “I think that one can draw useful conclusions for real life from this study,” and “I think that the methodology is fundamentally useless to investigate the effects of violent video games”) plus the two items “I think that the authors have no idea about video games” and “I think that the authors know a lot about violent video games” (recoded); (Cronbach’s α_confutative_ = .82; Cronbach’s α_confirmatory_ = .87). Response scales ranged from 1 (*not at all true*) to 6 (*very much true*). Higher values indicate more negative evaluations.

Finally, demographic information was assessed and participants were debriefed and thanked. Completing the survey took about 13 minutes. Descriptive statistics and correlations between the evaluation variables and identification are displayed in Table 5.

**Table 5:** Descriptive Statistics and Correlations between Variables in Study 2.

Variable	M (SD)	Correlations			
		(1)	(2)	(3)	(4)
Identification with the group of gamers (1)	2.87 (1.32)	1.00			
Negative evaluations of the confutative study (2)	3.44 (0.91)	−0.04	1.00		
Negative evaluations of the confirmatory study (3)	3.66 (1.02)	0.29***	0.15**	1.00	
Biased evaluations ^a^ (4)	0.22 (1.26)	0.27***	−0.60***	0.70***	1.00

*Notes*. *N* = 655.

^a^ Difference score: negative evaluations of the confirmatory study minus negative evaluations of the confutative study

*p < .05

**p < .01

***p < .001.

### Analytical Strategy and Content Analysis

**Liking/disliking and posting behavior**. The two central dependent variables, that is, liking/disliking and posting behavior, were analyzed via multinomial logistic regression analyses (MNLR) with identification as predictor. MNLR is suited for analyzing multiple qualitatively different categories of one dependent variable. In our case, response patterns coded with 0 served as the reference category (see Tables 3 and 4). Notably, identification with the group of gamers can have two separate effects on liking/disliking behavior: One effect refers to the question whether and to what degree identification can differentiate between participants who show a disliking bias against the confirmatory (and a liking bias in favor of the confutative) study compared to those who like both studies equally well. The other effect refers to the question whether and to what degree identification can differentiate between participants who show a liking bias in favor of the confirmatory (and a disliking bias against the confutative) study and those who like both studies equally well. As with liking/disliking there are also two effects of interest concerning posting behavior. The first effect is whether and to what degree identification can differentiate between participants who show a posting bias against the confirmatory (and in favor of the confutative) study and those who show no posting bias. The second effect refers to the question whether and to what degree identification can differentiate between participants who show a posting bias in favor of the confirmatory (and against the confirmatory) study and those who show no posting bias. We expected that identification with the group of gamers would predict a higher likelihood of a disliking bias and a posting bias against the confirmatory study (compared to liking both studies equally well) and would predict a lower likelihood of a liking bias and a posting bias in favor of the confirmatory study (compared to liking both studies equally well).

**Content analysis**. Posted comments were also content analyzed. There were a total of *N = *1120 comments (634 “contra” and 486 “pro” comments). Each relevant statement within a comment was coded according to the coding scheme shown in Table 6. All relevant statements were classified into one of five categories. The coding scheme was generated deductively upon our hypotheses and inductively by coding a subset of 10% (*n = *112) of the comments [47]. Two research assistants blind to the hypotheses were trained with a random sample of 15% of the comments (*n = *168). Thereafter, the two research assistants independently coded the remaining comments with a random overlap of 10% of all comments (*n = * 112), which were used to assess inter-rater reliability. The unit of analysis upon which reliability was determined was the frequency of the respective category coding for each comment. The inter-rater reliability, assessed by Krippendorff’s α [48], was satisfactory in all categories, all αs > .71 [49].

**Table 6:** Content Analysis Coding Scheme and Results Employed in Study 2.

Category (coding frequency)			Intercoder Reliability α	Example statement	Correlation with identification with the group of gamers (Spearman’s ρ)	
					Confutative study condition	Confirmatory study condition
Evaluative statement referring directly to the study	positively evaluative (*n* = 512)		.89	“This study seems to be very sound.”	.10**_a_	−.09*_b_
	negatively evaluative	reference to methodology (e.g., design, validity, etc.; *n* = 703)	.91	“The number of participants seems too low to draw conclusions for the population.”	−.05_a_	.16***_b_
		reference to other issues (e.g., competence of authors, relevance, conclusion; *n* = 375)	.92	“The conclusion is nonsense.”	.00_a_	.05_a_
Opinion statement on the effects of violent video games	violent video games have no detrimental/positive effects or statement relativizing detrimental effects (*n* = 258)		.94	“For me, violent video games are an outlet to release pressure.”	.14***_a_	.09*_a_
	violent video games have detrimental effects (*n* = 77)		.71	“Generally, I think that violent video games lead to aggressive behavior.”	−.09*_a_	−.03_a_

*Notes*. α denotes Krippendorff’s Alpha. Sample statements were translated from German. *N* = 655.

*p < .05

**p < .01

***p < .001. Correlation coefficients in the same row with no common lowercase subscript differ at *p* < .05 using Williams’ test.

For each participant category coding frequencies of each category were pooled across the “pro” and “contra” comment within each condition (resulting in one category coding frequency for each category in each condition, see Table 6). When a participant did not post a comment into the textbox the respective frequency was set to zero. Analyses were conducted by correlating each category coding frequency with the identification measure using Spearman’s *ρ*, since all coding frequencies were positively skewed (all skewness > 1.97). We expected that strong identifiers compared to weak identifiers would write more negative evaluative statements in the confirmatory condition and that this is not the case in the confutative study condition. The correlation coefficients were compared using Williams’ test [50]. With regard to the effect opinion statements, we did not expect a difference in the confirmatory compared to the confutative study condition for strong identifiers compared to weak identifiers.

### Results

**Liking/Disliking Behavior**. Identification positively predicted a disliking bias against the confirmatory (and in favor of the confutative) study, *B* = .38, *SE* = .07, *p* < .001, *OR* = 1.46, 95% CI [1.28, 1.68], and negatively predicted a liking bias in favor of the confirmatory (and against the confutative) study, *B* = −.35, *SE* = .10, *p* < .001, *OR* = 0.70, 95% CI [0.58, 0.85]. In other words, strongly identified gamers are more likely to show a disliking bias against and less likely to show a liking bias in favor of the confirmatory study compared to weakly identified gamers.

**Figure 2:**
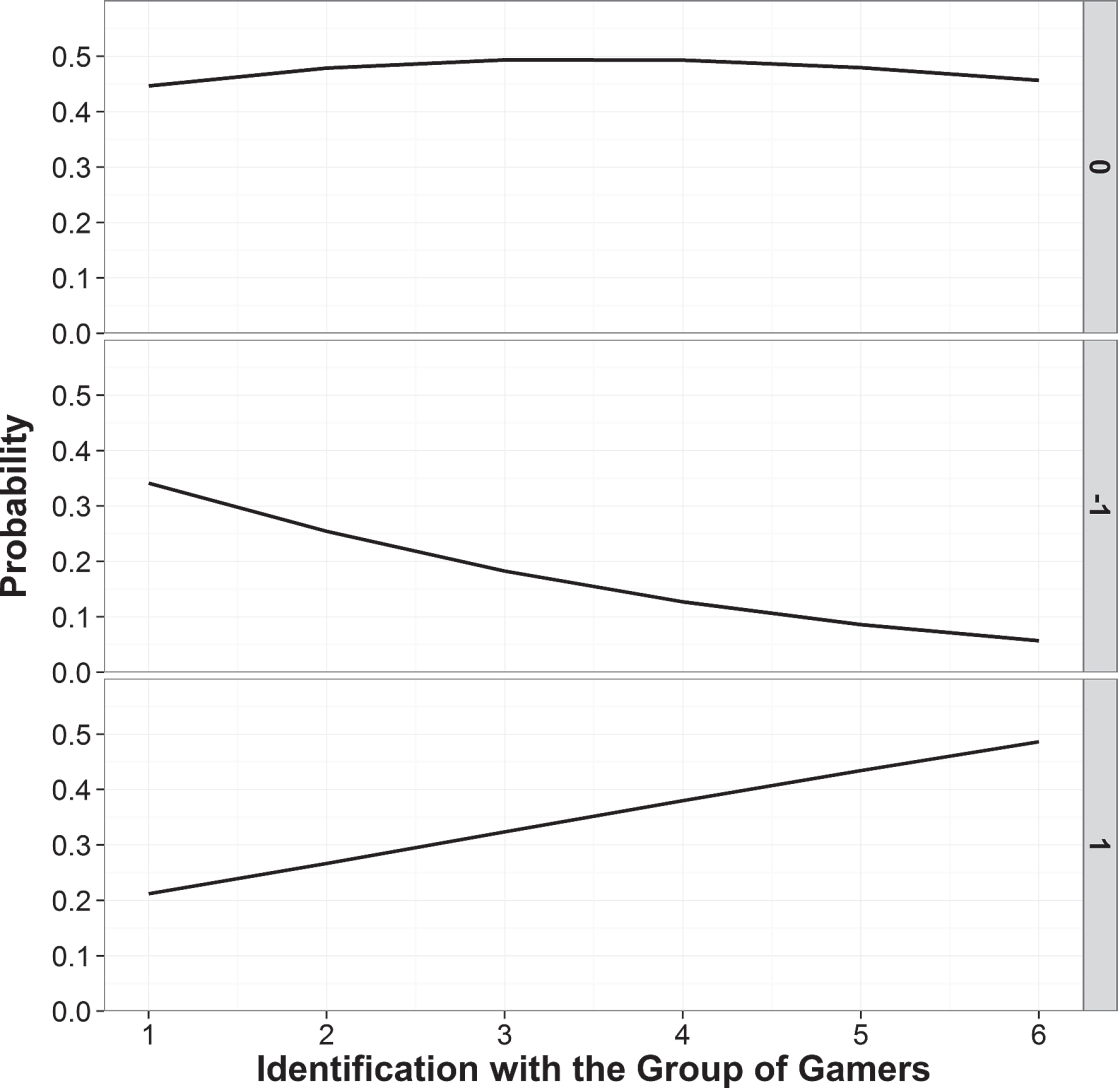
Probability of a posting bias against the confirmatory study (“1”), a posting bias in favor of the confirmatory study (“-1”) and no bias (“0”) as a function of identification with the group of gamers predicted by MNLR (Study 2).

**Posting Behavior**. Mirroring the results regarding liking/disliking behavior, identification positively predicted a posting bias against the confirmatory (and in favor of the confutative) study, *B* = .16, *SE* = .07, *p* = .02, *OR* = 1.12, 95% CI [1.03, 1.34], and negatively predicted a posting bias in favor of the confirmatory (and against the confutative) study, *B* = −.36, *SE* = .09, *p* < .001, *OR* = 0.70, 95% CI [0.59, 0.83]. In other words, strongly identified gamers (compared to weakly identified gamers) were more likely to show a posting bias against the confirmative study and less likely to show a posting bias in favor of the confirmatory study. The effects are displayed in Fig. 2.

**Content Analysis**. As can been seen in Table 6, identification with the group of gamers negatively predicted the number of positive evaluative statements, *r_s_* = −.09, *p* = .02, 95% CI [-.16, -.01] (*r_s_* denotes Spearman’s *Rho*), and positively predicted the number of negative evaluative statements criticizing the methodology, *r_s_* = .16, *p* < .001, 95% CI [.09, .24], in the confirmatory study condition. Both effects were significantly different from their counterparts in the confutative study condition, *t*s > 3.07, *p*s < .003. In other words, strong identifiers less often praised the confirmatory study and methodologically criticized it more heavily than weak identifiers did. In contrast to the evaluative statements, identification did not differently predict the effect opinion statements in both conditions, *t*s < 1.01, *p*s > .30. In both conditions strong identifiers more often stated a no-effect opinion, *r_s_confutative_* = .14, *p* < .001, 95% CI [.06, .21], *r_s_confirmatory_* = .09, *p* = .03, 95% CI [.01, .16], and less often stated a detrimental-effect opinion *r_s_confutative_* = −.09, *p* = .02, 95% CI [-.17, -.01], *r_s_confirmatory_* = −.03, *p* = .45, 95% CI [-.10, .05]. In sum, the results of the content analysis qualify the effects found on posting behavior and demonstrate that strong identifiers particularly criticized the methodology and denied to write positive statements about a potentially threatening study within their comments.

**Evaluations**. In general, the confirmatory study was evaluated more negatively (*M* = 3.66, *SD* = 1.02) than the confutative study (*M* = 3.44, *SD* = 0.91), *t*(654) = 4.50, *p* < .001, *d* = .18, 95% CI [.10, .25] [51]. Notably, the bias against the confirmatory study (i.e., the difference between the negative evaluations of the confirmatory study and the negative evaluations of the confutative study) was predicted by the identification with the group of gamers, *r_p_* = .27, p < .001, 95% CI [.20, .34] (*r_p_* denotes Pearson’s *r*). More specifically, the stronger participants identified with the group of gamers, the more negatively they evaluated the confirmatory study, *r_p_confirmatory_* = .29, *p* < .001, 95% CI [.22, .36], whereas identification did not predict the evaluation of the confutative study, *r_p_confutative_* = −.04, *p* = .26, 95% CI [-.12, .03].

### Discussion

The results of Study 2 mirror and extend our findings from Study 1. They provide additional support for our notion that strongly identified gamers are more inclined to take action against research when this research constitutes a social identity threat. When confronted with research findings that corroborate the violent-games-effect hypothesis, strongly identified gamers reacted with more “dislikes” and negative comments towards the respective research. Notably, strong identifiers particularly criticized the methodology of the confirmatory study. Furthermore, strongly identified gamers evaluated the confirmatory study more negatively than weakly identified gamers, replicating earlier findings of Nauroth and colleagues [11]. Whereas Study 2 investigated whether research that implies a social identity threat leads to science-discrediting behavioral responses from strongly identified group members, Study 3 tested whether this effect can be alleviated by alternatively affirming one’s social identity.

## Study 3

Study 3 aimed at investigating the psychological mechanism that explains why strongly identified group members are more likely to comment negatively on research that potentially threatens their social identity. Since strong identifiers’ self-concept is more heavily based on their group membership, a social identity threat has more profound consequences for their self-concept [14,19], which, in turn, should lead to a higher motivation to restore their social identity [29]. In other words, we hypothesize that posting a negative comment in response to potentially threatening research is instrumental for restoring a positive social identity. If our reasoning was true, then achieving the same goal, that is, to restore and affirm one’s social identity, via alternative means should alleviate the tendency to post negative comments. In line with this assumption, previous research has already shown that collective affirmation increases the acceptance of group-threatening information [32]. Thus, if posting negative comments actually aimed at restoring one’s social identity, such posting behavior should become less likely when one’s social identity has already been affirmed otherwise.

Importantly, this hypothesis is not trivial: research by Derks and colleagues [33] showed that collectively affirming group members does not alter their motivation to act collectively against a social identity threat. These authors showed that collectively affirming group members subjectively transformed the threat into a challenge and experiencing a challenge to one’s social identity does not reduce collective action tendencies. This stands in contrast to our reasoning predicting a decreased tendency to act collectively after being collectively affirmed. However, the study by Derks and colleagues differs in some important aspects from the present study. Most importantly, they investigated this effect only in the performance motivation domain, in which it is reasonable to assume that a threat appraisal can be restructured into a challenge appraisal [52]. It is thus an open question whether a social identity threat emanating from scientific findings in a public debate can also be restructured in a challenge appraisal when group members were collectively affirmed. If this was the case, a collective affirmation should not lead to a decreased tendency for collective action for strong identifiers. However, if our reasoning was true, then strong identifiers should be less inclined to post a discrediting comment after being collectively affirmed.

Another alternative explanation could be that posting negative comments is motivated by personal identity threat [26]. Gamers, particularly strong identifiers, may feel personally attacked by research showing that violent video games have detrimental consequences, and these gamers may be motivated to post a negative comment in order to liberate them from the personal deprecation that accompanied such research. In other words, posting negative comments may not so much be a collective, but rather a personal issue. The present study was designed to test these two competing hypothesis against each other.

### Method

**Participants**. The study was advertised in several German university student mailing lists (excluding the one used in Study 2). In all, 632 people started the questionnaire; 512 of them (80%) finished it successfully. Fifty-three participants were excluded from further analyses because these people indicated that they had not played any video games during the last 12 months (*n* = 45) or had more than 25% missing values on the identification measure (*n* = 8). Therefore, the final sample consisted of 459 participants (39% women). Ages ranged between 18 and 50 years (*M* = 23.89; *SD* = 3.91). One tablet computer was raffled among all participants who completed the survey.

**Procedure and Measures**. We used the same cover story as in Study 2: participants were told that a new blog about new research findings would be launched soon, that the first topic of this new blog would be violent video games, and that the present survey was designed to find out which studies are best suited to be presented in this blog. After this introduction, identification with the group of gamers was measured with the same items used in Study 2 (Cronbach’s α = .92; *M* = 2.82, *SD* = 1.28).

**Collective vs. Self-Affirmation Manipulation**. Next, participants were told that the evaluation of scientific results was affected by one’s “verbal-linguistic” competence and that we therefore wanted to control for that influence (for a similar procedure, see Derks et al. [33]). Participants were asked to complete a short “verbal-linguistic” competence test, which consisted of ten anagrams that were designed to increase the likelihood that participants would feel that they were good at this. After the bogus test, participants were asked how difficult they perceived the test to be with two items (“I found the test to be difficult” and “I think that I scored well in the test”). Ratings were obtained on six-point scale (1 = *strongly disagree*, 6 = *strongly agree*). The means of both items departed significantly in the desired direction from the midpoint of the scale with *t*(452) = 20.57, *p* < .001, *M* = 2.46, and *t*(457) = 14.32, *p* < .001, *M* = 4.28.Then, participants read a summary of one study demonstrating aggression-enhancing effects of violent video games (behavioral aggression measure/confirmatory study from Study 2, see S1 Appendix). Afterwards, participants were randomly assigned to a collective affirmation, self-affirmation, or no affirmation control condition. In the *self-affirmation condition*, participants read that their personal performance on this test (relative to a representative German sample) was “above average.” In the *collective affirmation condition*, participants were informed that we were not able to give them personal feedback at this point, but that–on the basis of a German representative sample–the average performance of people who regularly play video games was “above average,” whereas the average performance of people who do not play video games was “below average”. In the *control condition* participants received no feedback. Importantly, identification with the group of gamers did not differ between the three conditions, *F*(2, 456) = 1.57, *p* = .21.

**Dependent Variable: Posting Behavior**. After the affirmation manipulation participants were asked to write a “pro” (positive) or “contra” (negative) comment about the study. Each response was coded into one of four response categories: only positive comment, only negative comment, both positive and negative comments, or no comment. Participants could also like/dislike the study and were asked for their evaluation of the study (same order and items as in Study 2). Since only identification with the group of gamers significantly predicted these variables in the same manner as in Study 2 for the confirmatory study condition, all *p*s < .001 and no significant condition or condition × identification interaction effects were found, all *p*s > .18, we refrained from presenting a detailed analysis here. However, these results replicate our findings from Study 2. We additionally content analyzed all comments in the same manner as in Study 2. We did not find any significant condition or condition × identification interaction effects on the content analytical categories, all *p*s > .15, and therefore also refrain from presenting a detailed analysis. However, the correlational pattern across the three conditions generally replicates our findings from Study 2 in the confirmatory study condition (see S3 Table). Finally, demographic information was assessed and participants were debriefed and thanked. Completing the survey took about 14 minutes.

**Analytical Strategy**. In order to analyze posting behavior, we again conducted a MNLR. Only “contra” comments were coded as +1, only “pro” comments were coded as –1, and no comments and/or “pro” *and* “contra” comments were coded as 0. The latter category served as the reference category. We expected that identification with the group of gamers positively predicts negative posting behavior in the control and self-affirmation condition, but not in the collective affirmation condition. Statistically speaking, we expected an affirmation × identification interaction effect. The three experimental conditions were effect-coded with *effect1* representing the difference between the self-affirmation condition and the grand mean (effect1: self-affirmation = 1, collective affirmation = 0, control = −1), and *effect2* representing the difference between the collective affirmation condition and the grand mean (effect2: self-affirmation = 0, collective affirmation = 1, control = −1), and identification was centered prior to computing product terms [43]. Simple effects of significant interaction effects were tested by conducting binary logistic regressions for the respective condition in contrast to the control condition (i.e., collective affirmation vs. control or self-affirmation vs. control) with identification as the predictor and posting behavior as the dependent variable (i.e., negative posting behavior vs. reference category or positive posting behavior vs. reference category).

### Results

**Figure 3:**
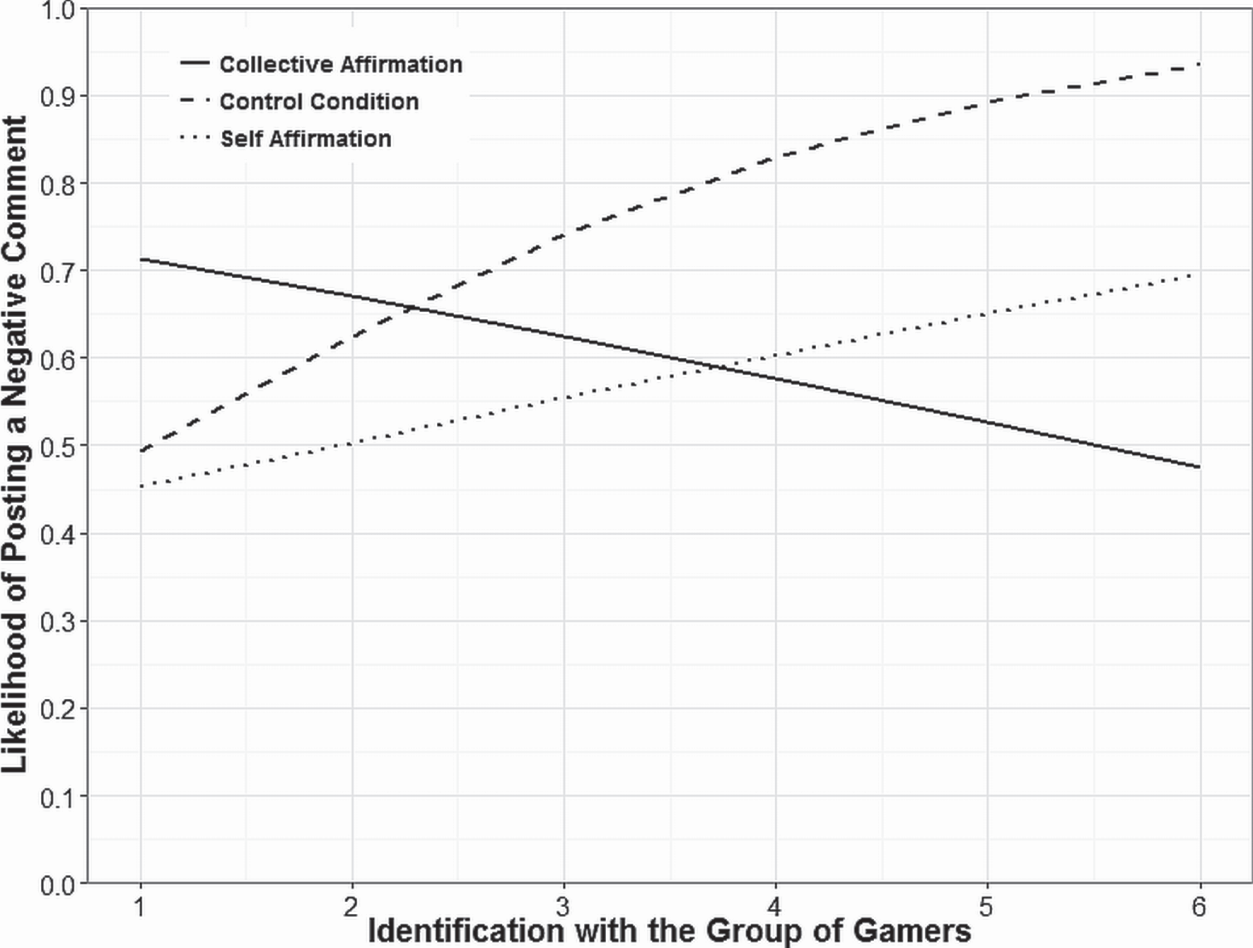
Likelihood of posting a negative comment against the confirmatory study vs. reference category (negative and positive or no comment) by identification with the group of gamers by condition predicted by binary logistic regressions (Study 3).

First, we tested the full model with posting behavior as the criterion and identification (mean-centered), effect1, effect2, identification × effect1, and identification × effect2 as predictors. Positive posting behavior was only predicted by identification, *B* = −.32, *SE* = .12, *p* = .009, *OR* = 0.73, 95% CI [0.58, 0.93]: the stronger the identification with the group of gamers, the lower the likelihood of posting a positive comment, irrespective of the affirmation condition. No other effects were significant on the 5% level. Negative posting behavior was also predicted by identification, *B* = .19, *SE* = .09, *p* = .045, *OR* = 1.20, 95% CI [1.00, 1.44] and by effect1 (the self-affirmation effect), *B* = −.38, *SE* = .16, *p* = .02, *OR* = 0.69, 95% CI [0.50, 0.93]. Strongly identified participants were more likely to post a negative comment than weakly identified participants; additionally, self-affirmed participants were less likely to post a negative comment than non-affirmed participants. However, the effect of identification was qualified by the hypothesized identification × effect2 interaction effect, *B* = −.39, *SE* = .12, *p* = .001, *OR* = 0.67, 95% CI [0.53, 0.86] (see Table 7). Simple slopes analysis was conducted via binary logistic regressions. As expected, the effect of identification was significant in the control condition, *B* = .54, *SE* = .18, *p* = .003, *OR* = 1.71, 95% CI [1.20, 2.42], but not in the collective affirmation condition, *B* = −.20, *SE* = .14, *p* = .15, *OR* = 0.82, 95% CI [0.62, 1.08]. In other words, strong identifiers were more likely to post a negative comment in the control condition, thus replicating the results of Study 2. However, when collectively affirmed, strong identifiers were not more likely to post a negative comment about the confirmatory study anymore. Importantly, the identification × effect1 interaction effect was not significant (*p* = .91), which implies that self-affirmation did not alleviate strong identifiers’ likelihood of posting a negative comment about the study. The effect of identification on negative posting behavior in each of the three affirmation conditions is depicted in Fig. 3.

**Table 7:** Results of Multinomial Logistic Regression Analysis (Study 3).

	Posting behavior	
	Positive	Negative
Intercept	−0.37*	0.56***
Identification with the group of gamers	−0.32**	0.18*
Effect1	−0.34	−0.38*
Effect2	−0.23	−0.01
Identification × Effect1	−0.08	0.01
Identification × Effect2	−0.33	−0.39**

*Notes*. *N* = 459.

*p < .05

**p < .01

***p < .001. Reference category: no posting or positive and negative posting.

### Discussion

Results from Study 3 replicate and extend the findings from Study 2. As in Study 2, strong identifiers were more likely than weak identifiers to post a negative comment against a potentially threatening research finding. However, when collectively affirmed, identification did no longer predict posting behavior. This finding provides evidence that writing a science-discrediting comment serves an affirmative goal: by writing a critical and negative comment against a threatening research finding, strong identifiers aim to restore their devalued social identity. Self-affirmation, on the other hand, did not interact with identification. However, self-affirmation led to a decrease in negative comments regardless of the participants’ degree of identification, thereby replicating a common finding from the self-affirmation literature [28]. This strengthens our argument that a social identity threat triggers a need for affirmation–particularly for strong identifiers–and that posting a negative comment appeases the need for affirmation. Importantly, we were able to show that–in the case of a social identity threat emanating from scientific findings–collectively affirming group members actually decreases collective action tendencies. This stands in contrast to the finding by Derks and colleagues [33] who found no effect of collective affirmation on collective action tendencies in the domain of performance and achievement. Thus, the effect of a collective affirmation on collective action tendencies seems to depend on the threat domain and on the type of collective action (online vs. offline).

## General Discussion

Research on science communication has grown during recent years. This research has mainly focused on people’s attitudes toward science and influencing factors. Much less research has been devoted to the question of whether the same factors explaining people’s attitudes also lead to behavioral consequences and how such behavioral inclinations can be motivationally explained. The present research aimed to fill this gap. We focused on posting science-discrediting online comments as a possible behavioral response toward social identity threat. The three studies reported in this article provide consistent support for our theoretical argument that posting science-discrediting comments in the online realm can be explained by social identity theory. All three studies consistently indicate that strongly identified group members more often write science-discrediting comments when the research is threatening to their social identity. Furthermore, Study 2 and 3 revealed that strong identifiers particularly aimed at impairing the credibility in the methodology of potentially threatening research. Finally, Study 3 demonstrated that posting negative and critical comments against threatening research is motivated by a need to restore one’s social identity.

These findings provide evidence for our assumption that research, when it implies a threat to one’s social identity, elicits critical communication among strongly identified group members in Internet forums, social networks, or online discussions, and that this communication is directed at restoring a positive social identity. On a theoretical level, our research provides new insight into the processes and factors that promote science-opposing actions by laypersons. In cases in which groups are the focus of research, our findings suggests that it is not only the quality of research that leads people to publicly criticize certain research results, but also the implications of the research for one’s social identity in conjunction with the degree to which people feel connected to the in-group. On an applied level, our research has immediate implications for the question which factors undermine the societal acceptance of scientific findings and which factors may motivate people to actively oppose such findings.

### Social Identity and Online Collective Behavior

The discharge of negative and hostile opinions on the Internet is often attributed to disinhibition effects of the online environment [53] or simply to an epidemic opinion spreading in the online social network cluster [54,55]. Our research challenges these perspectives and suggests that posting science-discrediting online comments can be regarded a form of collective action. The SIDE model and social identity theory offer immediate and well-founded predictions for online (collective) behavior. As shown in all three studies, people were motivated to write a discrediting comment when they identified strongly with their in-group, which demonstrates that identity concerns are an important factor in order to understand online behavior. Concerning individual behavior, this perspective is supported by Toma and Hancock [29]. These authors showed that people used Facebook for self-affirmative concerns after their ego was threatened. Our results conceptually extend Toma and Hancock’s findings by showing that after a social identity threat people also make use of the respective online features in order to collectively affirm their social identity (Study 3).

However, we would not argue that identity concerns are the only motivation behind science discrediting behavior. Being collectively affirmed did indeed diminish the likelihood to discredit science, but it did not eliminate it entirely (see Fig. 3). Other motivations might be particularly influential when the respective scientific result has immediate implications for policy making (i.e., in socio-scientific debates). For example, in the case of the violent video games debate, strong identifiers might also fear sale bans or accessibility restrictions being based upon research demonstrating a games-aggression link. In this case posting a negative comment might be also motivated by the intention to prevent research findings from affecting policy. Whereas immediate social identity concerns are possibly the spontaneous motivational component of discrediting threatening science, trying to prevent research results from affecting policies might be the more strategic component. In fact, strategic considerations have been shown to be very influential for how people express their social identity in public (see [56]). Thus, future research might investigate which other factors, besides social identity concerns, motivate science-discrediting behavior.

The new features of the Web 2.0 also give rise to the question whether and to what degree online collective action is truly something qualitatively different then offline collective action. In order to conceptually extend the typology of online collective action, Van Laer and Van Aelst [57] distinguished between low-threshold (i.e., low-risk, low-cost) collective actions (e.g., joining and liking a group’s Facebook page, signing online petition, posting comments) and high-threshold (i.e., high-risk, high-cost) collective actions (e.g., “hacktivism,” establishing and maintaining a protest website). Whereas it seems reasonable to assume that high-threshold online collective action and classical offline collective action are more likely to have similar underlying mechanisms [25] low-threshold online collective action is different. For instance, Schuman [58] demonstrated that low-threshold online collective action actually impedes offline collective action: people who participate in low-threshold online collective action are less likely to participate in offline collective action, whereas this was not the case for high-threshold online collective action. Furthermore, low-threshold online collective action might also be generally perceived as less effective than offline collective action [59] implying that the effect of efficacy as predictor of collective action tendencies [17] might be modulated by the particular setting. These and other issues are of high interest for theory building in collective action research since it seems likely that low-threshold online collective action is going to become more frequent the more people make use of the new communicational features of Web 2.0.

### Limitations and Suggestions for Future Research

The present research dealt with the violent video games debate. By focusing on one debate, we cannot be sure whether our conclusions are applicable to other cases in which groups are affected by research results as well. However, since we based our predictions on general principles of the social identity theory (i.e., social identity threat and social identification), which have been shown to be applicable to a whole range of social groups and intergroup phenomena [60], our results should also be applicable to other instances in which social groups are affected by research. Nonetheless, future research should investigate our predictions in other instances in order to test whether our findings can be generalized beyond the violent video games debate. Other examples in which our research results might be applicable are men discrediting research on sexism, unionists on research investigating potential adverse effects of unions on the economy, or vegetarians questioning the validity of research showing negative health effects of a vegetarian diet.

Our findings provide first evidence for social identity concerns not only influencing individual attitudes towards science, but furthermore motivating science-discrediting behavior on the Internet (and elsewhere). We will now discuss two open questions, which arise from our findings, and we hope that this discussion will stimulate further research on these topics.

**Target of the communication**. In the experimental setup of Studies 2 and 3, participants were told that the studies were conducted in order to identify suitable scientific studies for an online blog. One could expect that the content, tone, or negativity of the comment changes as a function of whom it is directed at: the public (or third parties), the out-group, or the in-group [56]. Comments directed at the public or third-parties should be particularly persuasive (i.e., calling the credibility of the research into question) in order to prevent others from getting a negative impression about the in-group (like in our studies). In contrast, comments directed at the out-group (in our case, laypersons and scientists arguing that violent video games are harmful) should be particularly derogative and offensive (cf. the example cited at the beginning of this article), whereas an in-group directed comment should aim at instigating collective action [22]. The latter case is particularly interesting since recent research shows that online comments effectively influence other in-groups members’ evaluation of information on the Web. Walther and colleagues [61] demonstrated that people who watched a YouTube video were more strongly influenced by the valence of the posted comments toward this video when they identified strongly with the peers posting the comments. This finding indicates that posting online comments can be an effective way to influence other in-group members’ opinions. From a collective action perspective, this finding suggests that online comments can be effective in motivating other in-group members to take action against threatening information. This also resonates with work by Postmes and Brunstig [59] who showed that activists perceived the mobilizing potential of the Internet as its biggest advantage. Thus, it would be worthwhile to investigate whether the content, tone, or negativity of the comment changes as a function of the target of the comment.

**Escalation of negative posts**. A somewhat related question that future research should address is how the escalation of negative posts sometimes observed in social networks and Web forums (“online firestorm,” [55]) can be explained. Why do some people write derogatory online comments even though several people had already posted a similar comment on the same issue before? These phenomena are particularly interesting since they stand in contrast to research findings suggesting that online social influence effects rather lead to a positivity bias and not to negative downward spirals [62]. However, if posting a comment was not only motivated by the desire to express one’s opinion, but rather by supporting the in-group, particularly strong identifiers should be prone to post negative comments even though others had done so already [63]. Like demonstrating on the street is more influential the more protesters are involved, posting discrediting online comments might also be perceived as more influential the more commentators are joining in. This interpretation is also in accord with findings that the higher the perceived efficacy of a collective action, the more likely collective action is taken [17]. This might even be enhanced by an expectancy that other users react affirmatively towards one’s negative comment when the group consensus was clearly and numerously expressed before. Thus, potential positive reactions from other commentators towards one’s own comment might be an additional affirmative source (besides the affirmative effect of one’s own comment). It would therefore be interesting to test these predictions in a simulated online environment in which people have the possibility to post comments and to react directly toward comments made by other participants.

### Science Communication and a Public Engagement with Science

The findings of this article contribute to the science communication literature in various ways. Most importantly, our findings offer a theoretical explanation for why certain scientific findings sometimes face a broad societal opposition: science-discrediting comments made by in-group members might persuade others to dismiss scientific findings as invalid [64,65]. Computer-mediated word-of-mouth has been shown to be highly persuasive [66,67]. In a real-life scenario of the cover story employed in Studies 2 and 3, the science-discrediting comments made by strongly identified group members might have persuaded the blog editors to dismiss the threatening articles from the blog. Such a persuading effect should be particularly intended when the comment is specifically directed at the general public or third-parties (see above). Notably, Anderson and colleagues [3] found that negative blog comments indeed have a persuading effect on laypersons’ perception of scientific findings and technologies. These authors found that uncivil blog comments polarized risk perceptions of nanotechnology depending on readers’ support for nanotechnology. Uncivil blog comments increased the risk perception of people opposing this technology and decreased the risk perception for people who were in favor of it. The findings of Anderson and colleagues therefore suggest that negative comments can impair the credibility of a scientific finding or an emerging technology. Thus, science-discrediting comments might actually lead parts of the public to question the research’s credibility and validity.

### Conclusions

The present research sheds light on why people post science-discrediting comments in blogs, social networks, and Web forums. In three experiments, we showed that identification with the group affected by research findings increases the likelihood to post a science-discrediting comment when the finding is potentially threatening to one’s social identity, and that these comments were aimed at reaffirming the threatened social identity. Theoretically, our research shows that the social identity approach is useful to explain hostile behavior in the online realm and the public engagement with science. On a more applied level, our research demonstrates why certain scientific findings sometimes face a broad societal opposition.

## Supporting Information

S1 Appendix Summaries used in Study 2. (DOCX)

S1 Table Separate Contrast Analysis for the Confutative and the Confirmatory Study Condition with Identification as the Dependent Variable and Posting Behavior as the Independent Variable. (DOCX)

S2 Table Separate Contrast Analysis for the Confutative and the Confirmatory Study Condition with Identification as the Dependent Variable and Liking/Disliking Behavior as the Independent Variable. (DOCX)

S3 Table Content Analysis Coding Scheme and Results Employed in Study 3. (DOCX)

S1 Data Datasets of Study 2 and Study 3. (ZIP)

## References

[1] Batts S, Anthis NJ, Smith T (2008) Advancing science through conversations: Bridging the gap between blogs and the academy. PLOS Biol 6: 1837–1841. 10.1371/journal.pbio.0060240 PMC2553846 18816167

[2] Metzger MJ, Flanagin AJ, Medders RB (2010) Social and heuristic approaches to credibility evaluation online. J Commun 60: 413–439. 10.1111/j.1460-2466.2010.01488.x

[3] Anderson AA, Brossard D, Scheufele DA, Xenos MA, Ladwig P (2014) The “nasty effect”: Online incivility and risk perceptions of emerging technologies. J Comput Mediat Commun 19: 373–387. 10.1111/jcc4.12009

[4] Bushman BJ (09 May 2011) Violent video games harm children. Available: http://www.youtube.com/watch?v=X3SA0YdK53g. Accessed 2014 Mar 31.

[5] Lewandowsky S, Ecker U, Seifert C, Schwarz N, Cook J (2012) Misinformation and its correction, continued influence and successful debiasing. Psychol Sci Public Interest 13: 106–131. 10.1177/1529100612451018 26173286

[6] Greitemeyer T (2014) I Am Right, You Are Wrong: How Biased Assimilation Increases the Perceived Gap between Believers and Skeptics of Violent Video Game Effects. PLOS ONE 9: e93440 10.1371/journal.pone.0093440 24722467 PMC3983102

[7] Lord CG, Ross L, Lepper MR (1979) Biased assimilation and attitude polarization: The effects of prior theories on subsequently considered evidence. J Pers Soc Psychol 37: 2098–2109. 10.1037/0022-3514.37.11.2098

[8] Munro GD (2010) The scientific impotence excuse: Discounting belief-threatening scientific abstracts. J Appl Soc Psychol 40: 579–600. 10.1111/j.1559-1816.2010.00588.x

[9] Scheufele D, Corley E, Shih T, Dalrymple KE, Ho SS (2008) Religious beliefs and public attitudes toward nanotechnology in Europe and the United States. Nat Nanotechnol 4: 91–94. 10.1038/nnano.2008.361 19197309

[10] Morton TA, Haslam SA, Postmes T, Ryan MK (2006) We value what values us: The appeal of identity-affirming science. Polit Psychol 27: 823–838. 10.1111/j.1467-9221.2006.00539.x

[11] Nauroth P, Gollwitzer M, Bender J, Rothmund T (2014) Gamers against science: The case of the violent video games debate. Eur J Soc Psychol 44: 104–116. 10.1002/ejsp.1998

[12] Ryan MK, Haslam SA, Postmes T (2007) Reactions to the glass cliff: Gender differences in the explanations for the precariousness of women’s leadership positions. J Organ Change Manag 20: 182–197. 10.1108/09534810710724748

[13] Glasman LR, Albarracin D (2006) Forming attitudes that predict future behavior: A meta-analysis of the attitude-behavior relation. Psychol Bull 132: 778–822. 10.1037/0033-2909.132.5.778 16910754 PMC4815429

[14] Tajfel H, Turner JJC (1979) An integrative theory of intergroup conflict. In: Austin WG, Worschel S, editors. The Social Psychology of Intergroup Relations (Vol. 33). Monterey, CA: Brooks/Cole pp. 33–47.

[15] Tajfel H, Turner JC (1986) The social identity theory of intergroup behavior. In: Worchel S, Austin WG, editors. The Psychology of Intergroup Relations. Chicago: Nelson-Hall pp. 7–24.

[16] Reicher SD, Spears R, Postmes T (1995) A social identity model of deindividuation phenomena. In Stroebe W, Hewstone M, editors. European Review of Social Psychology (Vol. 6). Chichester, UK: Wiley pp. 161–198. 10.1080/14792779443000049

[17] Van Zomeren M, Postmes T, Spears R (2008) Toward an integrative social identity model of collective action: A quantitative research synthesis of three socio-psychological perspectives. Psychol Bull 134: 504–535. 10.1037/0033-2909.134.4.504 18605818

[18] Turner J, Hogg M, Oakes P, Reicher S, Wetherell M (1987) Rediscovering the social group: A self-categorization theory. Oxford, UK: Blackwell.

[19] Wood W (2000) Attitude change: persuasion and social influence. Annu Rev Psychol 51: 539–570. 10.1146/annurev.psych.51.1.539 10751980

[20] Branscombe NR, Ellemers N, Spears R, Doosje B (1999) The context and content of social identity threat. In: Ellemers N, Spears R, Doosje B, editors. Social identity: Context, commitment, content. Oxford, UK: Blackwell pp. 35–58.

[21] De Hoog (2013) Processing of social identity threats: A defense motivation perspective. Soc Psychol 44: 361–372. 10.1027/1864-9335/a000133

[22] Scheepers D, Spears R, Doosje B, Manstead ASR (2003) Two functions of verbal intergroup discrimination: Identity and instrumental motives as a result of group identification and threat. Pers Soc Psychol Bull 29: 568–577. 10.1177/0146167203029005002 15272991

[23] Spears R, Lea M, Postmes T (2007) Computer-mediated communication and social identity. In: Joinson A, McKenna K, Postmes T, Reips U, editors. Oxford Handbook of Internet Psychology. Oxford: Oxford University Press.

[24] Postmes T, Spears R, Sakhel K, De Groot D (2001) Social influence in computer-mediated communication: The effects of anonymity on group behavior. Pers Soc Psychol Bull 27: 1243–1254. 10.1177/01461672012710001

[25] Postmes T (2007) The psychological dimensions of collective action, online. In: Joinson A, McKenna K, Postmes T, Reips U-D, editors. Oxford Handbook of Internet Psychology. Oxford, UK: Oxford University Press pp. 165–184.

[26] Sherman DAK, Nelson LD, Steele CM (2000) Do messages about health risks threaten the self? Increasing the acceptance of threatening health messages via self- affirmation. Pers Soc Psychol Bull 26: 1046–1058. 10.1177/01461672002611003

[27] Steele CM (1988) The psychology of self-affirmation: Sustaining the integrity of the self. In: Berkowitz L, editor. Advances in Experimental Social Psychology (Vol. 21). New York: Academic Press pp. 261–302

[28] Sherman DK, Cohen GL (2006) The psychology of self-defense: Self-affirmation theory. In Zanna MP, editor. Advances in experimental social psychology (Vol. 38). San Diego, CA: Academic Press pp. 183–242. 10.1016/S0065-2601(06)38004-5

[29] Toma CT, Hancock JT (2013) Self-affirmation underlies Facebook use. Pers Soc Psychol Bull 39: 321–331. 10.1177/0146167212474694 23359086

[30] Glasford D, Dovidio J, Pratto F (2009) I continue to feel so good about us: In-group identification and the use of social identity-enhancing strategies to reduce intragroup dissonance. Pers Soc Psychol Bull 35: 415–427. 10.1177/0146167208329216 19141621

[31] Derks B, Van Laar C, Ellemers N (2006) Striving for success in outgroup settings: Effects of contextually emphasizing ingroup dimensions on stigmatized group members’ social identity and performance styles. Pers Soc Psychol Bull 32: 576–588. 10.1177/0146167205283336 16702152

[32] Sherman DK, Kinias Z, Major B, Kim HS, Prenovost M (2007) The group as a resource: Reducing biased attributions for group success and failure via group affirmation. Pers Soc Psychol Bull 33: 1100–1112. 10.1177/0146167207303027 17630262

[33] Derks B, Van Laar C, Ellemers N (2009) Working for the self or working for the group: How self- versus group affirmation affects collective behavior in low-status groups. J Pers Soc Psychol 96: 183–202. 10.1037/a0013068 19210074

[34] Bushman BJ, Huesmann LR (2013) Twenty-five years of research on violence in digital games and aggression revisited: A reply to Elson & Ferguson (2013) Eur Psychol 19: 47–55. 10.1027/1016-9040/a000164

[35] Elson M, Ferguson CJ (2014) Twenty-five years of research on violence in digital games and aggression: Empirical evidence, perspectives, and a debate gone astray. Eur Psychol 19: 33–46. 10.1027/1016-9040/a000147.

[36] Pollard Sacks D, Bushman BJ, Anderson CA (2011) Do violent video games harm children? Comparing the scientific amicus curiae “experts” in Brown v. Entertainment Merchants Association. Northwestern University Law Review: Colloquy 106: 1–12. Available: http://www.law.northwestern.edu/lawreview/colloquy/2011/15/LRColl2011n15PollardSacks.pdf.

[37] Przybylski AK (2014) Who believes electronic games cause real-world aggression? Cyberpsychol Behav Soc Netw 17: 228–234. 10.1089/cyber.2013.0245 24256132

[38] Sjöström A, Sowka A, Gollwitzer M, Klimmt C, Rothmund T (2013) Exploring audience judgments of social science in media discourse: The case of the violent video games debate. Journal of Media Psychology 25: 27–38. 10.1027/1864-1105/a000077

[39] Leach CW, Van Zomeren M, Zebel S, Vliek MLW, Pennekamp SF, et al (2008) Group-level self-definition and self-investment: A hierarchical (multicomponent) model of in-group identification. J Pers Soc Psychol 95: 144–165. 10.1037/0022-3514.95.1.144 18605857

[40] Mael FA, Tetrick LE (1992) Identifying organizational identiﬁcation. Educ Psychol Meas 52: 813–824. 10.1177/0013164492052004002

[41] Ashmore RD, Deaux K, McLaughlin-Volpe T (2004) An organizing framework for collective identity: Articulation and significance of multidimensionality. Psychol Bull 130: 80–114. 10.1037/0033-2909.130.1.80 14717651

[42] Cohen J, Cohen P, West S, Aiken L (2003) Applied multiple regression/correlation analysis for the behavioral sciences (3rd ed.). Mahwah, NJ: Lawrence Erlbaum Associates Inc

[43] Aiken L, West S (1991) Multiple regression: Testing and interpreting interactions. Newbury Park, CA: Sage.

[44] Schwarz N (1999) Self-reports: How the questions shape the answers. Am Psychol 54: 93–105. 10.1037/0003-066X.54.2.93

[45] Wilson RE, Gosling SD, Graham LT (2012). A review of Facebook research in the social sciences. Perspect Psychol Sci 7: 203–220. 10.1177/1745691612442904 26168459

[46] Postmes T, Haslam SA, Jans L (2013) A single-item measure of social identification: Reliability, validity, and utility. Br J Soc Psychol 52: 597–617. 10.1111/bjso.12006 23121468

[47] Früh W (2007) Inhaltsanalyse: Theorie und Praxis (6st ed). Konstanz: UVK.

[48] Hayes AF, Krippendorff K (2007) Answering the call for a standard reliability measure for coding data. Commun Methods Meas 1: 77–89. 10.1080/19312450709336664

[49] Krippendorff K (2004). Reliability in content analysis: Some common misconceptions and recommendations. Hum Commun Res 30: 411–433. 10.1111/j.1468-2958.2004.tb00738.x

[50] Steiger JH (1980) Tests for comparing elements of a correlation matrix. Psychol Bull 87: 245–251. 10.1037/0033-2909.87.2.245

[51] Kelley K (2007) Confidence intervals for standardized effect sizes: Theory, application, and implementation. Journal of Statistical Software 20(8): 1–24. Available: http://www.jstatsoft.org/v20/i08/.

[52] Blascovich J, Tomaka J (1996) The biopsychosocial model of arousal regulation. In: Zanna MP, editor. Advances in experimental social psychology (Vol. 28). San Diego, CA: Academic Press pp. 1–51.

[53] Suler J (2004) The online disinhibition effect. Cyberpsychol Behav 7: 321–326. 10.1089/1094931041291295 15257832

[54] Kramer ADI, Guillory JE, Hancock JT (2014) Experimental evidence of massive- scale emotional contagion through social networks. Proc Natl Acad Sci U S A 111: 8788–8790. 10.1073/pnas.1320040111 24889601 PMC4066473

[55] Pfeffer J, Zorbach T, Carley KM (2014) Understanding online ﬁrestorms: Negative word-of-mouth dynamics in social media networks. J Market Comm 20: 117–128. 10.1080/13527266.2013.797778

[56] Klein O, Spears R, Reicher S (2007) Social identity performance: Extending the strategic side of the SIDE model. Pers Soc Psychol Rev 11: 28–45. 10.1177/1088868306294588 18453454

[57] Van Laer J, Van Aelst P (2010) Internet and social movement action repertoires. Information, Communication & Society 13: 1146–1171. 10.1016/j.cmpb.2014.12.003 25562877

[58] Schuman S (2014) Click to Act? The (De)Mobilizing Effect of Expressive Low-threshold Online Collective Actions: Motivational Underpinnings and Contextual Boundaries. Doctoral Thesis, Université Libre de Bruxelles.

[59] Brunstig S, Postmes T (2002) Social movement participation in the Digital Age: Predicting offline and online collective action. Small Group Res 33: 525–554. 10.1177/104649602237169

[60] Abrams D, Hogg MA (1999) Social identity and social cognition. Oxford, England: Blackwell

[61] Walther JB, DeAndrea D, Kim J, Anthony JC (2010) The influence of online comments on perceptions of antimarijuana public service announcements on YouTube. Hum Commun Res 36: 469–492. 10.1111/j.1468-2958.2010.01384.x

[62] Muchnik L, Aral S, Taylor JT (2013) Social influence bias: A randomized experiment. Science 341: 647–651. 10.1126/science.1240466 23929980

[63] Jackson JW (2011) Intragroup cooperation as a function of group performance and group identity. Group Dyn 15: 343–356. 10.1037/a0024575

[64] Houston JB, Hansen GJ, Nisbett GS (2013) Influence of user comments on perceptions of media bias and third-person effect in online news. ENews 7: 79–92. 10.1177/1931243111407618

[65] Huesmann LR, Taylor LD (2003) The case against the case against media violence. In: Gentile D, editor. Media Violence and Children. Westport: CT: Greenwood Press pp. 107–130

[66] Edwards C, Edwards A, Qing Q, Wahl ST (2007) The influence of computer-mediated word-of-mouth communication on student perceptions of instructors and attitudes toward learning course content. Commun Educ 56: 255–277. 10.1080/03634520701236866

[67] Walther JB, Van Der Heide B, Hamel LM, Shulman HC (2009) Self-generated versus other-generated statements and impressions in computer-mediated communication: A test of warranting theory using Facebook. Communic Res 36: 229–253. 10.1177/0093650208330251

