# Supplementary material for: Social Identity Threat Motivates Science-Discrediting Online Comments
Source: PLoS One. 2015 Feb 3;10(2):e0117476. doi: 10.1371/journal.pone.0117476 (PMC4315604; doi:10.1371/journal.pone.0117476)
Supplement: S1 Appendix — (DOCX) [file pone.0117476.s001.docx]

## Summaries used in Study 2 (confutative study condition in squared brackets):

*Methodology 1:*

“In an experiment conducted by Jones and colleagues 227 participants were randomly assigned to one of two conditions. In one condition participants played a violent video game, participants in the other condition played a non-violent video game. After playing the video game, they were asked to participate in a reaction time task in which they competed (ostensibly) against an opponent seated in another room. Whenever participants won a round, they had the opportunity to punish the opponent. The punishment consisted of a very unpleasant noise (“white noise”); participants were asked to calibrate the sound’s duration and its volume; these settings served as measures of participants’ aggressive tendencies. Comparing the average duration and volume settings between the two conditions showed that those who had played the violent video game reacted [did not react] more aggressively than those who played the non-violent video game. The authors of the study concluded that consuming violent video games leads [does not lead] to an increase in aggression. The authors stated that “violent video games provide a forum for learning and practicing aggressive reactions“ [“playing violent video games does not constitute a risk factor for behaving aggressively“].”

*Methodology 2:*

“Kroner and colleagues investigated the effects of violent video games with functional magnetic resonance tomography (fMRI) in order to scrutinize the potential aggression-enhancing effects of violent video games on the brain. FMRI allows picturing the physiological features of the brain and the arousal level of the respective brain areas. In their experiment 13 male regular video game players played a violent video game. Brain activity was recorded via fMRI during the entire experiment and analyzed on an image-by-image basis. Participants’ in-game behavior was categorized as either „passive“ or „violent”. This categorization was compared to fMRI signals. Brain areas that are involved in real-life aggression had been identified in previous studies.

Results showed that these areas were particularly active during gameplay. These activity patterns were not [also] observed in game sequences in which no violent behavior took place. This finding indicates that virtual violence activates [does not activate] the same brain areas as real life violence.

The authors of the study hypothesize that this effect reflects a long-term outcome of a regular exposure to violent video games [is particularly the case for regular video game players] because all participants had violent video games experience. They warn against the negative effects of violent video games. [They conclude that violent video games have no negative effects.]
