# Supplementary material for: Social Identity Threat Motivates Science-Discrediting Online Comments
Source: PLoS One. 2015 Feb 3;10(2):e0117476. doi: 10.1371/journal.pone.0117476 (PMC4315604; doi:10.1371/journal.pone.0117476)
Supplement: S1 Table — (DOCX) [file pone.0117476.s002.docx]

## Table S1

*Separate Contrast Analysis for the Confutative and the Confirmatory Study Condition with Identification as the Dependent Variable and Posting Behavior as the Independent Variable.*

| Confutative Study | | | | |
| --- | --- | --- | --- | --- |
| Posting Behavior | *M (SD)* of Identification | Contrast Analysis | | |
|  |  | Contrast1 | Contrast2 | Contrast3 |
| only positive | 3.12 (1.26) | 3 | 0 | 0 |
| positive and negative | 3.10 (1.46) | -1 | 2 | 0 |
| no comment | 2.75 (1.43) | -1 | -1 | 1 |
| only negative | 2.65 (1.20) | -1 | -1 | -1 |
|  | *t*-values | 2.47^*^ | 1.95 | 0.70 |
|  |  |  | | |
| Confirmatory Study | | | | |
| Posting Behavior | *M (SD)* of Identification | Contrast Analysis | | |
|  |  | Contrast1 | Contrast2 | Contrast3 |
| only positive | 2.44 (1.19) | -1 | -1 | -1 |
| no comment | 2.75 (1.44) | -1 | -1 | 1 |
| positive and negative | 3.20 (1.51) | -1 | 2 | 0 |
| only negative | 3.10 (1.22) | 3 | 0 | 0 |
|  | *t*-values | 2.69^**^ | 2.72^**^ | 1.96 |
| *Notes.* *N* = 655. ^*^*p* < .05; ^**^*p* < .01; ^***^*p* < .001. | | | | |
