# Supplementary material for: Social Identity Threat Motivates Science-Discrediting Online Comments
Source: PLoS One. 2015 Feb 3;10(2):e0117476. doi: 10.1371/journal.pone.0117476 (PMC4315604; doi:10.1371/journal.pone.0117476)
Supplement: S2 Table — (DOCX) [file pone.0117476.s003.docx]

## Table S2

*Separate Contrast Analysis for the Confutative and the Confirmatory Study Condition with Identification as the Dependent Variable and Liking/Disliking Behavior as the Independent Variable.*

| Confutative Study | | | |
| --- | --- | --- | --- |
| Liking/Disliking Behavior | *M (SD)* of Identification | Contrast Analysis | |
|  |  | Contrast1 | Contrast2 |
| positive | 3.18 (1.32) | 2 | 0 |
| none | 2.57 (1.26) | -1 | -1 |
| negative | 2.62 (1.27) | -1 | 1 |
|  | *t*-values | 5.64^***^ | 0.39 |
|  |  |  | |
| Confirmatory Study | | | |
| Liking/Disliking Behavior | *M (SD)* of Identification | Contrast Analysis | |
|  |  | Contrast1 | Contrast2 |
| positive | 2.49 (1.31) | -1 | 1 |
| none | 2.54 (1.22) | -1 | -1 |
| negative | 3.31 (1.25) | 2 | 0 |
|  | *t*-values | 7.97^***^ | 0.42 |
| *Notes.* *N* = 655. ^*^*p* < .05; ^**^*p* < .01; ^***^*p* < .001. | | | |
