# Supplementary material for: Social Identity Threat Motivates Science-Discrediting Online Comments
Source: PLoS One. 2015 Feb 3;10(2):e0117476. doi: 10.1371/journal.pone.0117476 (PMC4315604; doi:10.1371/journal.pone.0117476)
Supplement: S3 Table — (DOCX) [file pone.0117476.s004.docx]

## Table S3

*Content Analysis Coding Scheme and Results Employed in Study 3*

| Category  (coding frequency) | | | Intercoder Reliability α | Correlation with identification with the group of gamers (Spearman’s *ρ*) |  |
| --- | --- | --- | --- | --- | --- |
|  |  |  |  |  |  |
| Evaluative statement referring directly to the study | positively evaluative (*n* = 143) | | 0.70 | -0.16^**^ |  |
|  | negatively evaluative | reference to methodology (e.g., design, validity, etc.; *n* = 279) | 0.85 | 0.15^**^ |  |
|  |  | reference to other issues (e.g.,  competence of authors, relevance, conclusion; *n* = 121) | 0.70 | 0.10^*^ |  |
| Opinion statement on the effects of violent video games | violent video games have no detrimental/positive effects or statement relativizing detrimental effects (*n* = 76) | | 0.85 | 0.11^*^ |  |
|  | violent video games have detrimental effects (*n* = 10) | | 1.00 | -0.03 |  |
| *Notes.* α denotes Krippendorff’s Alpha. Sample statements were translated from German. *N* = 459. ^*^*p* < .05; ^**^*p* < .01; ^***^*p* < .001. | | | | |  |
